# Comparison of 2 Natural Language Processing Methods for Identification of Bleeding Among Critically Ill Patients

**DOI:** 10.1001/jamanetworkopen.2018.3451

**Published:** 2018-10-12

**Authors:** Maxwell Taggart, Wendy W. Chapman, Benjamin A. Steinberg, Shane Ruckel, Arianna Pregenzer-Wenzler, Yishuai Du, Jeffrey Ferraro, Brian T. Bucher, Donald M. Lloyd-Jones, Matthew T. Rondina, Rashmee U. Shah

**Affiliations:** 1 Department of Biomedical Informatics, University of Utah School of Medicine, Salt Lake City; 2 Division of Cardiovascular Medicine, University of Utah School of Medicine, Salt Lake City; 3 Department of Internal Medicine, University of Utah School of Medicine, Salt Lake City; 4 Division of Pediatric Surgery, University of Utah School of Medicine, Salt Lake City; 5 Department of Preventive Medicine, Northwestern University Feinberg School of Medicine, Chicago, Illinois; 6 George E. Wahlen Veterans Affairs Medical Center Geriatric Research Education and Clinical Center, Salt Lake City, Utah; 7 Molecular Medicine Program, University of Utah School of Medicine, Salt Lake City

## Abstract

**Importance:**

To improve patient safety, health care systems need reliable methods to detect adverse events in large patient populations. Events are often described in clinical notes, rather than structured data, which make them difficult to identify on a large scale.

**Objective:**

To develop and compare 2 natural language processing methods, a rules-based approach and a machine learning (ML) approach, for identifying bleeding events in clinical notes.

**Design, Setting, and Participants:**

This diagnostic study used deidentified notes from the Medical Information Mart for Intensive Care, which spans 2001 to 2012. A training set of 990 notes and a test set of 660 notes were randomly selected. Physicians classified each note as *present* or *absent* for a clinically relevant bleeding event during the hospitalization. A bleeding dictionary was developed for the rules-based approach; bleeding mentions were then aggregated to arrive at a classification for each note. Three ML models (support vector machine, extra trees, and convolutional neural network) were developed and trained using the 990-note training set. Another instance of each ML model was also trained on a sample of 450 notes, with equal numbers of bleeding-present and bleeding-absent notes. The notes were represented using term frequency–inverse document frequency vectors and global vectors for word representation.

**Main Outcomes and Measures:**

The main outcomes were accuracy, sensitivity, specificity, positive predictive value, and negative predictive value for each model. Following training, the models were tested on the test set and sensitivities were compared using a McNemar test.

**Results:**

The 990-note training set represented 769 patients (296 [38.5%] female; mean [SD] age, 67.42 [14.7] years). The 660-note test set represented 527 patients (211 [40.0%] female; mean [SD] age, 67.86 [14.7] years). Bleeding was present in 146 notes (22.1%). The extra trees down-sampled model and rules-based approaches were similarly sensitive (93.8% vs 91.1%; difference, 2.7%; 95% CI, −3.8% to 7.9%; *P* = .44). The positive predictive value for the extra trees model, however, was 48.6%. The rules-based model had the best performance overall, with 84.6% specificity, 62.7% positive predictive value, and 97.1% negative predictive value.

**Conclusions and Relevance:**

Bleeding is a common complication in health care, and these results demonstrate an automated and scalable detection method. The rules-based natural language processing approach, compared with ML, had the best performance in identifying bleeding, with high sensitivity and negative predictive value.

## Key Points

**Question:**

Can a natural language processing approach that uses text from clinical notes identify bleeding events among critically ill patients?

**Findings:**

In this diagnostic study of a rules-based natural language processing model to identify bleeding events using clinical notes, the model was superior to a machine learning approach, with high sensitivity and negative predictive value. The extra trees machine learning model had high sensitivity but poor positive predictive value.

**Meaning:**

Bleeding complications can be detected with a high-throughput natural language processing algorithm, an approach that can be used for quality improvement and prevention programs.

## Teaser

This diagnostic study compares a rules-based natural language processing (NLP) model with a machine learning (ML) model to detect bleeding events among patients in an intensive care unit (ICU) using clinical notes.

## Introduction

Bleeding is a common complication in health care and is associated with increased morbidity, mortality, and health care costs.^1,2^ Big data approaches, incorporating genetic, sociodemographic, medical, and environmental exposures, provide an opportunity to create powerful models to predict which patients are likely to bleed. The challenge is finding bleeding events—the patient outcome required to create big data models to predict bleeding—across thousands, or even millions, of patients. An accurate, high-throughput method to identify bleeding is a missing prerequisite to develop new approaches to reduce harm.

Current methods to identify bleeding events rely on billing codes, which lack accuracy,^3,4^ or manual medical record review, which is not scalable. Clinical notes are now commonly in electronic form and are an underused data source. Progress notes, history and physical notes, and discharge summaries are generated as part of routine clinical care and often include narrative descriptions of bleeding events, such as *right femoral hematoma,* or explicitly mention the absence of bleeding, such as *patient denies bleeding.* The goal of this study was to develop and compare different natural language processing (NLP) algorithms for accurately identifying bleeding events in clinical notes. Natural language processing is a collection of computer-based methods for automatically locating such phrases and interpreting them to produce meaningful, computable data. In other words, clinicians read the medical record to find out whether their patients bled, but this approach cannot be easily scaled across entire health care systems. With NLP, the computer can “read” the note and extract bleeding events in a computable form for use in predictive models, quality and safety monitoring, and clinical decision support systems.

One common NLP approach, referred to as a rules-based (RB) approach, uses a list of physician-provided keywords and a predetermined set of rules to locate relevant text in a note and mark it as either *bleeding present* or *bleeding absent.* This list of keywords, often referred to as a knowledge base, includes the terms that represent bleeding in the text (*bleeding* or *hemorrhage*) and the modifier terms that create context (*denies* or *history of*). For example, 1 rule may be that if the computer locates the word *BRBPR* (bright red blood per rectum) in the text, it will mark the text as *bleeding present*, unless that word is preceded by the word *no* in the same sentence. In the latter case, the text would be marked as *bleeding absent.*

An alternative approach to identifying relevant health events in clinical text is to leverage machine learning (ML) techniques. Using this approach, an investigator produces a set of notes that have been labeled by physicians as having bleeding either present or absent. These notes are then analyzed using an ML algorithm to train a model that is capable of accurately applying bleeding-present or bleeding-absent labels to new notes. The goal of this study was to develop and compare RB and ML NLP algorithms to detect bleeding events.

## Methods

### Population

For this study, we used clinical notes from the Medical Information Mart for Intensive Care (MIMIC-III) database. This database is a publicly available, comprehensive, anonymized, clinical data set of intensive care unit (ICU) hospitalizations in a tertiary academic center.^5^ The database contains information on ICU encounters that occurred between 2001 and 2012 and represents real-world patients and real-world clinical practice. Our goal was to create a note-level classifier that could identify clinically relevant bleeding cases. We randomly selected 990 notes, irrespective of *International Classification of Diseases, Ninth Revision* (*ICD-9*), codes, to serve as the training set and 660 notes to serve as the test set. These notes represent the general adult ICU population, and the number of notes reviewed was based on time constraints. Each individual note was classified as *bleeding present,* meaning the clinically relevant bleeding was referenced in the note, or *bleeding absent,* meaning that clinically relevant bleeding was not referenced in the note. Clinically relevant bleeding was defined as symptomatic bleeding that required treatment.^6^ In less severe cases, we opted to classify cases as *present* if there was a change in clinical management. For example, if the note referenced bleeding from a central line, the note was marked as *bleeding present* if heparin was discontinued. We included history and physical notes, progress notes, and discharge summaries that were signed by an attending physician. This study used publicly available deidentified data and the authors determined that it did not require institutional review board review or patient informed consent. All investigators with direct access to the data completed a MIMIC data use agreement.

Two physicians (S.R. and A.P.-W.) independently classified each note as positive or negative for a clinically relevant bleeding event during the hospitalization. A third physician (R.U.S.) adjudicated disagreements. The physician classification served as the gold standard for comparison with the NLP-generated note-level classification. After 120 notes, interreviewer agreement was 93%, so we shifted to a single reviewer per note.

We created note-level bleeding detection algorithms rather than multinote or encounter-level algorithms. Development of the bleeding detection algorithms took place before the 660 test notes had been annotated to avoid biasing the development process with information from the test data.

### Rules-Based Classifier Development and Evaluation

To derive the knowledge base for the RB approach, we focused on a set of notes (separate from the training and test sets) that were rich in bleeding references by selecting hospitalizations that included *ICD-9* codes suggestive of bleeding (eg, 431, intracerebral hemorrhage). The corpus of notes from hospitalizations with an *ICD-9* code for bleeding was randomly parsed into batches of 30 for expert review using the Extensible Human Oracle Suite of Tools, a software tool that facilitates the document review process.^7^ Two physicians (R.U.S. and B.A.S.) reviewed the batches of notes and a third physician (B.T.B.) adjudicated discrepant annotations. Each specific reference to bleeding in the note was classified as *bleeding present* or *bleeding absent* (mention-level annotation). *Bleeding present* meant that the reference represented a clinically relevant bleeding event experienced by the patient during the hospitalization. *Bleeding absent* meant that the reference to bleeding was intended to exclude clinically relevant bleeding. The *absent* classification could include negated references (*patient denies bleeding*), historical references (*history of bleeding ulcer*), or hypothetical references (*transfuse if patient bleeds*). The purpose of the mention-level annotation was to provide a representative set of examples of how clinically relevant and nonrelevant bleeding cases are expressed in clinical notes. This step is necessary for rule development in RB NLP systems.

We used pyConText, a freely available software package that is based on the ConText algorithm, for the RB NLP task.^8,9^ Other open source NLP packages exist^10,11,12^ (eg, cTAKES^11^ [clinical Text Analysis and Knowledge Extraction System]), but are more complex, including part-of-speech tagging, section demarcation, and other features. Our goal was to make a simple, lightweight system that can be understood by clinicians for a single task. The pyConText package employs user-defined target (*bleeding, hemorrhage,* etc) and modifier (*denies, reports,* etc) terms to determine mention-level classifications. For example, a user would provide the knowledge that *bleeding* is a target and that *denies* is a negation modifier term. If both these terms are in the same span, as in *patient denies bleeding,* pyConText would use a rule to classify the mention as *bleeding absent.* We iteratively updated the NLP rules to improve algorithm test characteristics in the training set (sensitivity, specificity, positive predictive value [PPV], and negative predictive value [NPV]).

The pyConText package provides mention-level classification of bleeding references in clinical notes, but our goal was to establish note-level classification using NLP. To this end, we used receiver operating curves to evaluate heuristics for converting the mention-level classifications produced by pyConText to a single document-level classification that could be compared with the gold standard. From the output of pyConText, 3 quantities were used to create classification heuristics: (1) the number of *bleeding present* mentions, (2) the number of *bleeding absent* mentions, and (3) the total number of mentions. We identified the heuristic that yielded the highest sensitivity and used it to produce document-level classifications for each note.

### Machine Learning Classifier Development and Evaluation

The ML strategy adopted for this study is known as supervised learning. Under the supervised-learning paradigm, data are labeled using a gold standard (eg, physician review) and then an ML algorithm is executed on those data to discern patterns that can be used to accurately label future data. We first converted the text into a feature space, or a numerical representation that can be processed by ML algorithms. We used 2 conversion methods known as *bag-of-words representation* and *word embeddings*^13^ *representation* for this study.

We trained 3 ML models for this study: support vector machine (SVM), extra trees (ET) classifier, and a convolutional neural network (CNN) model. The SVM and ET models both used the bag-of-words feature representation. Each note was converted to a high-dimensional, bag-of-words vector in which each dimension was the term frequency–inverse document frequency (TF-IDF) score of all 1, 2, and 3 g (n-grams) in the note.^14^ We then used χ^2^ feature selection to select the features that had strong associations with bleeding. This resulted in a 100-dimensional representation of each note, in which each dimension represented 1 of the 100 most discriminatory n-grams. The hyperparameters of the SVM and ET were selected using a grid search with 10-fold cross-validation. In terms of a traditional multivariable prediction model, the variables for the SVM and ET models were effectively the IDF-weighted frequencies of the top 100 most characteristic n-grams.

To train the CNN, we used global vectors for word representation. The notes were represented as sequences of global vectors for word representation word embeddings rather than as 100-dimensional vectors of TF-IDF values.^15^ The hyperparameters of the CNN were selected using a fixed train validation split of 80/20. The CNN was constructed with 4 hidden layers, alternating between a 1-dimensional convolutional layer and a max pooling layer. The convolutional layers used a linear rectifying unit activation function. The output layer was a dense layer with a single unit and a sigmoid activation. The loss was calculated using binary cross-entropy.

To increase the sensitivity of the ML models, we also trained another instance of each model on a down-sampled data set. In this data set, the numbers of bleeding-present and bleeding-absent notes were equal, resulting in a down-sampled training set of 450 notes. In summary, 6 ML models were trained and evaluated: 3 on the full data set and 3 on the down-sampled data set.

### Statistical Analysis

After training, the 6 ML and RB models were evaluated on the 660-note test set. We calculated the sensitivity, specificity, PPV, *F* score, and NPV of each model compared with the reference standard. We selected the ML model with the highest sensitivity and compared it with the RB model using a McNemar test. A 2-sided *P* value of .05 was considered significant. This test calculates the likelihood that the results produced by 2 diagnostic tests are equivalent. In this case, it was used to compare the predictions of the best-performing ML model with those of the RB model. It produces a *P* value, similar to a traditional χ^2^ test. We opted to prioritize sensitivity, as bleeding events during hospitalization contribute to adverse outcomes, such as mortality, increased length of stay, and higher health care costs.

We used Python to develop the RB and ML algorithms. The code can be found at https://github.com/MaxTaggart/NLPBleedingDetection.

## Results

We manually reviewed 990 notes for the training set and 660 notes for the test set. The training set represented 769 patients, of whom 38.5% (296) were female. The mean (SD) age was 67.42 (14.7) years. The test set represented 527 patients, of whom 40.0% (211) were female. The mean (SD) age was 67.86 (14.7) years. The prevalence of clinically relevant bleeding was 22.5% in the training set and 22.1% (146 of 660) in the test set (Table 1).

**Table 1.** Patient Characteristics for Training and Testing Sets

Characteristic	Training Set	Test Set
Notes, No.	990	660
Unique patients, No.	769	527
Female, No. (%)	296 (38.5)	211 (40.0)
Age, mean (SD), y	67.42 (14.7)	67.86 (14.7)
Bleeding present, %	22.5	22.1

### Rules-Based Results

**Figure 1.**
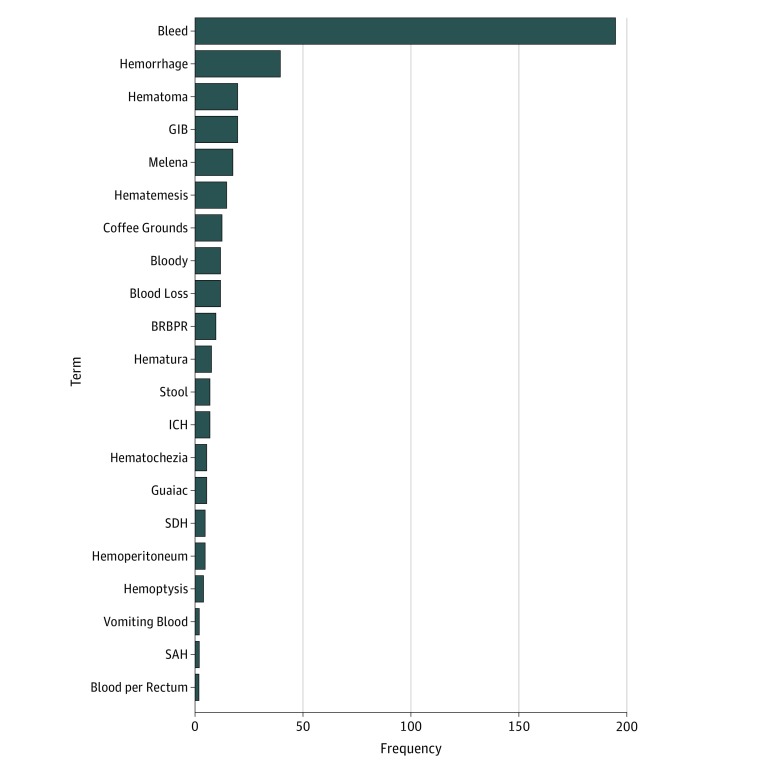
Frequency of Bleeding-Related Terms Automatically Identified by pyConText in 120 Clinical Notes Terms that are frequently used by clinicians to describe bleeding in clinical notes were identified during the knowledge generation phase and then used as target terms in the natural language processing algorithm. BRBPR indicates bright red blood per rectum; GIB, gastrointestinal bleeding; ICH, intracerebral hemorrhage; SAH, subarachnoid hemorrhage; and SDH, subdural hematoma.

In the knowledge generation phase, we reviewed 120 notes (distinct from the training and test sets) and identified a total of 406 bleeding mentions using 76 different terms. We aggregated the terms into 16 common stems (eg, *hemorr* for *hemorrhagic* and *hemorrhage*), which accounted for 90% of all 406 mentions. The most common term was *bleed,* followed by *hemorrhage* (Figure 1). We selected target and modifier terms from the knowledge development phase for use in pyConText.

**Figure 2.**
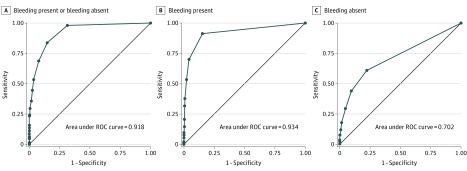
Receiver Operating Characteristic (ROC) Curves for Identifying Clinically Relevant Bleeding From Clinical Notes Using a Rules-Based Approach We evaluated 3 natural language processing–derived parameters to identify notes with clinically relevant bleeding in the training data for the total number of mentions (A), the number of bleeding-present mentions (B), and the number of bleeding-absent mentions (C). At least 1 absent or present reference to bleeding, identified by the algorithm, had almost 100% sensitivity for identifying notes with clinically relevant bleeding, but had poor specificity. At least 1 present reference to bleeding was 93% sensitive for identifying notes with clinically relevant bleeding, with greater than 70% specificity.

**Figure 3.**
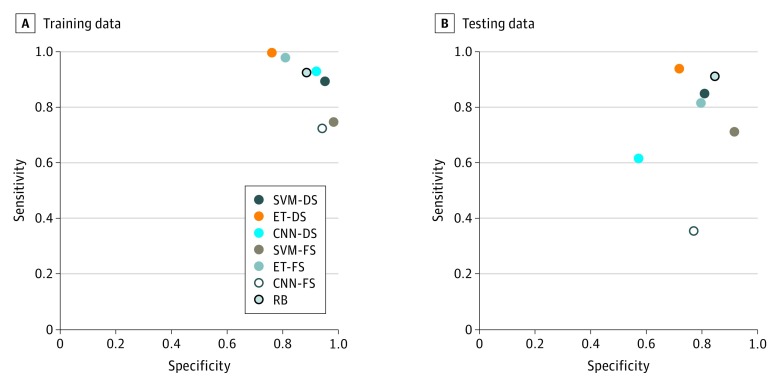
Test Characteristics for Different Computer Algorithms to Automatically Identify Clinically Relevant Bleeding Model names that include *-DS* (down-sampled) were trained on a note set with an equal number of present and absent events (n = 450) and then tested on the full 660-note test set. Those that include *-FS* (full sample) were trained on the full training set (n = 990) and then tested on the full 660-note test set. CNN indicates convolutional neural network; ET, extra trees; RB, rules based; SVM, support vector machine.

We applied the terms and modifiers from the knowledge generation phase to the 990 notes in the training set. In the first run, pyConText identified 1139 bleeding-present mentions and 614 bleeding-absent mentions in 990 notes. We performed error analyses, iteratively revised the rules, and reran the pyConText algorithm. After 3 cycles, we arrived at the final algorithm; the target and modifier terms, with regular expressions used in the program, are shown in eTables 1 and 2 in the Supplement. The *C* statistic was highest for number of bleeding-present mentions (*C* = 0.934) (Figure 2B), followed by overall total number of mentions (*C* = 0.918) (Figure 2A) and total number of bleeding-absent mentions (*C* = 0.702) (Figure 2C). Based on these results, we developed the following rule: if pyConText identifies 1 or more bleeding-present mention, classify the note as positive. The test characteristics for note classification in the training set using this rule were 92.4% sensitivity, 88.5% specificity, 70.3% PPV, and 97.6% NPV (Figure 3A).

### Machine Learning Results

In the training set, 243 870 bag-of-words features were present. After feature selection, we included 100 features associated with bleeding in the model. The top 20 features associated and not associated with bleeding are listed in eTable 3 in the Supplement). Each model was first evaluated on the training data. Models trained on the down-sampled data set are referred to in this article with a *-DS* suffix. The ET-DS model performed best on the training set, with 99.6% sensitivity, followed by the ET model (97.8% sensitivity) and the CNN-DS model (92.9% sensitivity). The CNN model performed the worst, with 72.4% sensitivity in the training set. In terms of PPV, the SVM-DS model performed best (Figure 3A; Table 2).

**Table 2.** Model Performance on the Training Set and Test Set for Identifying Clinically Relevant Bleeding

Model	Accuracy	Sensitivity	Positive Predictive Value	*F* Score^a^	Negative Predictive Value	Specificity
**Training Set (990 Notes)**						
SVM-DS	0.992^b^	0.893	0.948^b^	0.920	0.899	0.951
ET-DS	0.878	0.996^b^	0.806	0.891	0.994	0.760
CNN-DS	0.924	0.929	0.921	0.925^b^	0.928	0.920
SVM-FS	0.928	0.747	0.923	0.826	0.929	0.982^b^
ET-FS	0.847	0.978	0.601	0.745	0.992^b^	0.809
CNN-FS	0.892	0.724	0.784	0.753	0.921	0.941
Rules-based	0.894	0.924	0.703	0.798	0.976	0.885
**Test Set (660 Notes)**						
SVM-DS	0.818	0.849	0.559	0.674	0.950	0.809
ET-DS	0.767	0.938^b^	0.486	0.640	0.976^b^	0.718
CNN-DS	0.582	0.616	0.290	0.395	0.840	0.572
SVM-FS	0.871^b^	0.712	0.707^b^	0.710	0.918	0.916^b^
ET-FS	0.800	0.815	0.531	0.643	0.938	0.796
CNN-FS	0.679	0.356	0.306	0.329	0.808	0.770
Rules-based	0.861	0.911	0.627	0.743^b^	0.971	0.846

Abbreviations: CNN, convolutional neural network; DS, down-sampled (the model was trained on a note set with an equal number of present and absent events [n = 450] and then tested on the full 660-note test set); ET, extra trees; FS, full sample (the model was trained on the full training set [n = 990] and then tested on the full 660-note test set); SVM, support vector machine.

^a^ *F* score is defined as the harmonic mean of the sensitivity and positive predictive value.

^b^ Highest-performing model for each metric.

### Rules-Based Algorithm vs Machine Learning

After finalizing the models on the training data, we tested the models using the 660-note test set. The ET-DS and RB models yielded the highest sensitivities, 93.8% and 91.1%, respectively (difference, 2.7%; 95% CI, −3.8% to 7.9%; *P* = .44). The ET-DS model achieved 71.8% specificity, 48.6% PPV, and 97.6% NPV for classifying clinically relevant bleeding. By comparison, the RB method achieved 84.6% specificity, 62.7% PPV, and 97.1% NPV (Figure 3B; Table 2). The ET model had the fastest computational time, and the RB model had the slowest (eTable 4 in the Supplement).

In a post hoc error analysis, we reviewed 92 notes that were misclassified by the RB algorithm; we focused on the false-positives given the low PPV of the models. Of the 92 notes, 79 (85.9%) were false-positives when compared with the gold standard of medical record review. We reviewed the notes that were incorrectly classified as positive by the RB approach and found that 26 should have been classified as *bleeding present* in the initial medical record review. Example phrases that were found by the RB algorithm but not by the physician reviewer are listed in eTable 5 in the Supplement). Other sources of error included bleeding references in the medical history and allergy note sections incorrectly classified as *bleeding present.* After reclassifying the 26 mischaracterized notes, the performance characteristics for the RB approach were 95.8% sensitivity, 89.1% specificity, 74.5% PPV, and 98.4% NPV for the test set. The corresponding values for the ET-DS approach were 96.3% sensitivity, 82.4% specificity, 60.8% PPV, and 93.2% NPV.

## Discussion

Bleeding is common in health care, and is morbid, costly, and often difficult to capture in large clinical data sets. In this study, we developed ML and RB NLP systems that identified bleeding among critically ill patients with sensitivity of more than 90%. These results highlight the feasibility of a high-throughput NLP algorithm to identify bleeding cases, a necessary precursor for strategies to predict and reduce the incidence of bleeding. In fact, the NLP algorithm found bleeding events that were missed by human reviewers, which emphasizes that computer systems designed by humans may be better suited to identify bleeding events than humans alone.

We compared RB and ML approaches and found that the RB and ET-DS approaches had similarly high sensitivity, but RB had better PPV. The PPV of all models was suboptimal, meaning that several false-positive results remained. Given the variability in the models’ performance, different models may be best suited for different tasks. For example, the RB approach may be the best candidate for use in a system that identifies clinically relevant bleeding without any human review given that it achieved the highest *F* score. The high sensitivity and NPV of both the RB and ET-DS methods suggests that the algorithms developed in this study could significantly reduce the burden of medical record review required to identify health events. If this algorithm were, for example, implemented as a safety surveillance tool, 694 notes that were classified as *bleeding absent* would not require review and the review task would be decreased by 70%.

Bleeding is a major problem in the course of medical care, and can result from treatment with anticoagulant or antiplatelet agents or invasive procedures and surgery. Patients in the ICU with gastrointestinal bleeding, for example, have up to 4 times higher risk of death and 8 additional days in the ICU compared with nonbleeding patients.^2^ Data analytic methods, or big data, have the potential to combine genomic, sociodemographic, medical, mobile sensor, and environmental exposures to identify patient clusters that have high risk for bleeding. These data-driven approaches, however, require an automated, scalable method to identify bleeding events. Our study and prior work demonstrate that NLP can be used to find bleeding events from data that are readily available in the electronic medical record. Lee et al^16^ developed an NLP tool to find bleeding events among patients treated with clopidogrel with similar sensitivity to our results. Their work included patients with a billing code for bleeding, resulting in a bleeding prevalence of 85% in the training and test sets, whereas we included a more realistic population with lower bleeding prevalence.

### Limitations

The use of NLP in health care is challenging; notes are frequently ungrammatical and are often inconsistently formatted. Ambiguity is common: MS, for example, can mean mitral stenosis or multiple sclerosis. With the RB approach, only a portion of the complexity of natural language can be captured by a small set of rules. For example, we did not incorporate rules that identify the bleeding site (eg, gastrointestinal bleed or nose bleed).

The main reason why the ML models in this study did not perform well was the small training set. One inherent challenge for ML is its dependence on large numbers of training examples to learn useful patterns. Creation of de novo training data is time-consuming and expensive, especially in a health care setting where clinical expertise is often required to produce high-quality data. Rules-based systems still dominate the clinical NLP domain,^17^ with reasonable performance.^18^ Still, recent advances in the field of deep learning may address the challenges and provide a promising direction for future work in health care–related NLP.^19,20^

We chose to develop a tool to identify bleeding in single clinical notes, rather than over a full encounter or patient lifetime. We chose note-level classification because this approach can be implemented in near real time, as clinical notes are signed and closed more promptly than encounters. Near real-time implementation is also another advantage of NLP over structured billing codes. All notes for each unique patient are not represented in the data set, so comparison between NLP and *ICD-9* bleeding codes was not possible. In a post hoc analysis, however, we limited the testing set to patients with NLP-identified bleeding (n = 146). Among these patients, only 54.1% had 1 of 313 possible *ICD-9* codes for bleeding, consistent with low sensitivity of the approach based on *ICD-9*.

This algorithm was developed and tested using patients from a single institution’s ICU and requires validation in other patient settings and at other institutions. Race/ethnicity are not readily available in MIMIC. In addition, we used a binary classification: bleeding was present or absent. Future work can combine NLP with structured data (eg, hemoglobin or blood transfusions) to classify bleeding according to severity. Finally, the data represent real-world patients, but from a single institution. The code for these models is available online, and readers are welcome to externally validate the algorithms.

## Conclusions

Treatment-related harm, including bleeding, is a serious problem in health care. As invasive treatments and technologies evolve, health care systems and clinicians need to know who is helped and who is harmed by these interventions. An NLP approach can be used to identify bleeding complications (ie, who is harmed) and the algorithm can be applied to thousands, or millions, of patients without the addition of another EHR checkbox or problem list. This approach will enable data-driven comparative safety analyses to identify the right treatments for the right patients.

## Notes

Supplement. **eTable 1.** Features Selection for Machine Learning: n-grams Associated and Not Associated With Clinically Relevant Bleeding **eTable 2.** Target Terms Used in Natural Language Processing Task to Identify Bleeding From Clinical Notes **eTable 3.** Modifier Terms Used in Natural Language Processing Task to Identify Bleeding From Clinical Notes **eTable 4.** Average Computational Time for Each Natural Language Processing Approach **eTable 5.** “Bleeding Present” Phrases Identified by the Rules-Based Algorithm, but Not Identified by the Physician Reviewer
