# Supplementary material for: Comparison of 2 Natural Language Processing Methods for Identification of Bleeding Among Critically Ill Patients
Source: JAMA Netw Open. 2018 Oct 12;1(6):e183451. doi: 10.1001/jamanetworkopen.2018.3451 (PMC6324448; doi:10.1001/jamanetworkopen.2018.3451)
Supplement: Supplement. — eTable 1. Features Selection for Machine Learning: n-grams Associated and Not Associated With Clinically Relevant Bleeding eTable 2. Target Terms Used in Natural Language Processing Task to Identify Bleeding From Clinical Notes eTable 3. Modifier Terms Used in Natural Language Processing Task to Identify Bleeding From Clinical Notes eTable 4. Average Computational Time for Each Natural Language Processing Approach eTable 5. “Bleeding Present” Phrases Identified by the Rules-Based Algorithm, but Not Identified by the Physician Reviewer [file jamanetwopen-1-e183451-s001.pdf]

Supplementary Online Content

Taggart M, Chapman WW, Steinberg BA, et al. Comparison of 2 natural language processing methods for identification of bleeding among critically ill patients. *JAMA Netw Open*. 2018;1(6):e183451. doi:10.1001/jamanetworkopen.2018.3451

eTable 1. Features Selection for Machine Learning: n-grams Associated and Not Associated With Clinically Relevant Bleeding

eTable 2. Target Terms Used in Natural Language Processing Task to Identify Bleeding From Clinical Notes

eTable 3. Modifier Terms Used in Natural Language Processing Task to Identify Bleeding From Clinical Notes

eTable 4. Average Computational Time for Each Natural Language Processing Approach

eTable 5. "Bleeding Present" Phrases Identified by the Rules-Based Algorithm, but Not Identified by the Physician Reviewer

This supplementary material has been provided by the authors to give readers additional information about their work.

© 2018 Taggart M et al. *JAMA Network Open.*

eTable 1. Features selection for machine learning: n-grams associated and not associated with clinically relevant bleeding[a]

Features associated with “bleeding absent”	Features associated with “bleeding present”
labetolol mg	bleed
syringe	gi
mmhg cardiac output	gi bleed
hypertrophic	egd
change make medications	gib
start spironolactone	hematoma
history significant diabetes	gastrointestinal bleed
significant diabetes	hemorrhage
pna patient	subdural
drainage incision	bleed gib
day omeprazole	gi bleed gib
initial chest	ffp
position seat	bleed gi bleed
room follow	gastrointestinal bleed gi
sputum culture grow	bleed gi
small bilateral	units units units
spiriva inh	octreotide
chest tube placement	prbcs
blood base intuba	hematuria
lastname continue	units units
[a]Feature selection was performed using chi-squared tests. The chi-square value was zero for all features associated with “bleeding absent.” Chi-square values range from 1.626 to 9.062 for features associated with “bleeding present”	

© 2018 Taggart M et al. *JAMA Network Open.*

eTable 2. Target terms used in natural language processing task to identify bleeding from clinical notes.

<table>
<tr><th>Clinical Concept</th><th>Regular Expression</th></tr>
<tr><td>bleed</td><td>(?<!non)(?<!non)(?<!non-)(?<!re)(bleed(?!ing)|bleeding(?!\stime))</td></tr>
<tr><td>blood loss</td><td>blood loss</td></tr>
<tr><td>blood per rectum</td><td>blood per rectum</td></tr>
<tr><td>bloody</td><td>(?<!non-)(?<!non)(?<!non)bloody</td></tr>
<tr><td>brbpr</td><td>brbpr</td></tr>
<tr><td>coffee grounds</td><td>coffee[\-](ground|grounds)</td></tr>
<tr><td>ecchymosis</td><td>ecchymos[ie]s</td></tr>
<tr><td>epistaxis</td><td>epistaxis</td></tr>
<tr><td>exsanguination</td><td>exsanguination</td></tr>
<tr><td>gib</td><td>\bl?gib\b</td></tr>
<tr><td>guaiac</td><td>((\bg|gua?iac)([\-]|\s+)((pos(itive)?)|\+)|guaiac\(\+\))</td></tr>
<tr><td>hematemesis</td><td>hematem[a-z]+</td></tr>
<tr><td>hematochezia</td><td>hematochezia</td></tr>
<tr><td>hematoma</td><td>hematoma</td></tr>
<tr><td>hematuria</td><td>hematuria</td></tr>
<tr><td>hemoperitoneum</td><td>hemoperitoneum</td></tr>
<tr><td>hemoptysis</td><td>hemoptysis</td></tr>
<tr><td>hemorrhage</td><td>(?<!splinter\s)hem{1,2}or{1,2}h{1,2}age?</td></tr>
<tr><td>ich</td><td>\bich</td></tr>
<tr><td>melena</td><td>mel[ae]n(a|ic)</td></tr>
<tr><td>ng lavage post</td><td>(ng|ngt)\s+lavage\s+((positive)|(pos)|\+)</td></tr>
<tr><td>ng lavage pre</td><td>((positive)|(pos)|\+) (ng|ngt) lavage</td></tr>
<tr><td>occult blood</td><td>(fecal\s+occult(\s+blood)?|\bob|\bfob)\s+pos(itive)?</td></tr>
<tr><td>sah</td><td>sah</td></tr>
<tr><td>sdh</td><td>sdh</td></tr>
<tr><td>stool</td><td>(maroon|red)\s+(stool|bowel\s+movement|bm)</td></tr>
<tr><td>vomitting</td><td>vomit[a-z]* blood</td></tr>
</table>

© 2018 Taggart M et al. *JAMA Network Open.*

eTable 3. Modifier terms used in natural language processing task to identify bleeding from clinical notes. A minus-sign (-) in the Rule column indicates that the modifier marked its associated target term as bleeding-absent. A plus-sign (+) indicates that the modifier confirmed its target term as bleeding-present.

Clinical Concept	Regular Expression	Rule
ago	\bago\b	-
can result in	\bcan\s+result\s+in\b	-
cc	(cc:|chief complaint:)	+
ddx	ddx	-
deny	denies|denied|denying	-
did not show	did not (show|reveal)	-
episode	episode of	+
episodes	episodes of	+
found to have	found to have	+
here with	here (with|w\\\|w/)	+
history	((h/o)|(h\\o)|(hx ?of)|history|\bhx\b|pmh)	-
history of	history\s+of	-
if	\bif\b	-
in the past	\bin\s+the\s+past\b	-
monitor	monitor(for)?	-
negative	\bnegative\b	-
negative dash	\(-\)	-
never	\bnever\b	-
no	\bno\b	-
no evidence	no (evidence|e\\o|e/o)	-
no history	no ((h/o)|(h\\o)]|hx)	-
non	non(\s|-)	-
not	not\b	-
now with	now (with|w\\\|w/)	+
possible	possible	-
presenting	presenting* (with|w\\\|w/)	+
presents	present[s|e][d]* (with|w\\\|w/)	+
previous	previous	-
prior	\bprior\b	-
risk	risk of	-
rule out	(rule out|r\/o)	-
recent admission	recent\s+admission	-
suspicion	suspicion	-
transfuse	transfuse	-
unlikely	unlikely	-
unrevealing	unrevealing\s+for	-
versus	versus|vs	-
watch	watch for	-
without	((without)|(w\\o)|(w/o))(?!\scontrast)	-

© 2018 Taggart M et al. *JAMA Network Open.*

eTable 4. Average computational time[a] for each natural language processing approach.

Model	Notes per Second
SVM	42.72
ET	5818.42
CNN	122.16
RB	3.09

[a]Computational time if calculated as the number of notes each model is able to process per second, as an average over the 660-note test set. These tests were run on a machine with a 4-core intel i7 CPU and 16 gigabytes of RAM.

SVM=support vector machine; ET=extra trees classifier; CNN=convolutional neural network; RB=rule-based model

© 2018 Taggart M et al. *JAMA Network Open.*

eTable 5. “Bleeding present” phrases identified by the rules-based algorithm, but not identified by the physician reviewer

left upper extremity hematoma
with signs/symptoms of bleeding
loose melena
L second toe with hemtoma present
likely hemorrhage related to
bleed noted at cath site
coffee-ground emesis
guaiac + stools
Small hematoma
re-op for chest wall hematoma
diffuse ecchymoses, INR > 22.8
guaic + stools
persistent area of bleeding
bloody exudate
R groin with ecchymosis
Anemia, acute, secondary to blood loss (Hemorrhage, Bleeding)
stools were guaiac positive
excess amounts of postosperative bleeding
left parietal subgaleal hematoma present
line site bleeding
subdural hematoma on L
consistent with alveolar hemorrhage

© 2018 Taggart M et al. *JAMA Network Open.*
